# Prevalence and risk factors for genital high-risk human papillomavirus infection among women attending the out-patient clinics of a university teaching hospital in Lagos, Nigeria

**DOI:** 10.11604/pamj.2017.28.227.13979

**Published:** 2017-11-14

**Authors:** Kehinde Sharafadeen Okunade, Chidinma Magnus Nwogu, Ayodeji Ayotunde Oluwole, Rose Ihuoma Anorlu

**Affiliations:** 1 Department of Obstetrics & Gynaecology, Lagos University Teaching Hospital Lagos, Nigeria; 2 Department of Obstetrics & Gynaecology, College of Medicine, University of Lagos, Lagos, Nigeria

**Keywords:** Genital, genotypes, hrHPV, LUTH, PCR, vaccination

## Abstract

**Introduction:**

Cervical cancer is the second most common cancer among women in the developing countries and the seventh commonest cancer in the developed countries. Human papillomavirus (HPV) is now known to be the main factor in the aetiology of cervical cancer with over 99.7% of cases being associated with previous high risk HPV (hrHPV) infection. This study was aimed to determine the prevalence and risk factors for genital hrHPV infection among women attending the out-patient clinics of the Lagos University Teaching Hospital.

**Methods:**

This was a cross-sectional study involving a total of 200 women. Questionnaires were administered to collect data such as sociodemographic, reproductive and sexual histories. Endocervical swab samples were then taken from each participant. Samples were analyzed by polymerase chain reaction (PCR) using consensus primers targeted against the hrHPV viruses.

**Results:**

The prevalence of hrHPV in the study was 36.5%. The most predominant HPV subtypes were 31 (25.0%), 35 (8.0%) and 16 (3.5%) with the largest proportion (76.1%) of the tested samples being positive for only a single hrHPV subtype. The study showed statistically significant associations between early age at coitarche (P = 0.032) and increasing number of lifetime sexual partners (P = 0.001) with genital hrHPV infection.

**Conclusion:**

The prevalence of hrHPV was high in Lagos with the majority of test positive samples having only a single HPV genotype. We demonstrated early age of sexual debut and increasing number of lifetime sexual partners as the most important factors associated with genital hrHPV infection.

## Introduction

Human papillomavirus (HPV) is now known to be the main factor in the aetiology of cervical cancer with over 99.7% of cases being associated with previous oncogenic or high-risk human papillomavirus (hrHPV) infection. Therefore, since HPV infection is a sexually transmitted infection, cervical cancer is now identified as a sexually transmitted cancer by origin [1]. Cervical cancer is the second most common cancer among women in the developing countries and the seventh commonest cancer in the developed countries [2]. Over 500,000 new cases are seen yearly with over 80% of them being from the developing countries [2-4]. Worldwide, cervical cancer claims the lives of about 300,000 women annually with over 80% coming from the developing countries [2, 5]. It is the most common gynaecological cancer and a leading cause of cancer death in women in Nigeria [6] where it kills one woman every hour and over 9000 women every year [7]. HPV is the most common sexually transmitted virus and it is estimated that about 75% of sexually active women and men will acquire a genital HPV infection at some time in their lifetime [8]. Worldwide, approximately 20 million women are infected with the virus [9]. HPV infection is an epitheliotropic infection [10, 11]. Most infections are subclinical and will cause no physical symptoms, however, in some people subclinical infections will become clinical and may cause benign papillomas (such as warts (verrucae) or squamous cell papilloma), or cancers of the cervix, vulva, vagina, penis, oropharynx and anus [12]. Persistent infection with the high-risk or oncogenic strains of HPV is now known to be a necessary cause of cervical cancer [13, 14]. The prevalence of genital human papillomavirus infection in sub-Saharan Africa is considered to be among the highest in the world just like cervical cancer [14]. It was also observed in a pooled analysis on HPV prevalence surveys that the highest HPV prevalence was seen in Nigeria [15, 16]. This study was therefore aimed to determine the prevalence and distribution of high risk-HPV infection among women attending the out-patient clinics of the Lagos University Teaching Hospital (LUTH) and then identifies the likely predisposing factors to this potentially deadly infection.

## Methods

This was a cross-sectional study carried out among women seen at the cytology and gynaecology outpatient clinics of the Lagos University Teaching Hospital (LUTH) over a period of 6 months. LUTH is an over 1000 bedded teaching hospital located in the Central Lagos metropolis in South-West Nigeria. The hospital immediate environ is inhabited by civil servants, students, traders and artisans. It also provides services to patients from the neighbouring South-Western states. The hospital is the largest in the state and offers mainly clinical services among which include gynaecological oncology services [17]. The gynaecology clinic is an all-female clinic with the cytology clinic being an off-shoot of it. In addition to receiving patients from the gynaecology clinic, the cytology clinic is the meeting point for all women from all other clinics of the hospital referred for routine cytological evaluation. The sample size for the study was determined using the statistical formula for cross-sectional descriptive study [18]. A total number of 200 women were selected by consecutive sampling method for the study. The participants were given information leaflet and counselled appropriately about the objective and methodology of the study. A written informed consent is obtained from each participant upon enrollment into the study. Excluded from the study were virgins, pregnant women, those who have undergone hysterectomy, women with obvious cervical lesions and those who were mentally or physically unable to undergo a pelvic examination. Questionnaires were administered to relevant data such as sociodemographic, reproductive and sexual histories.

Endocervical swab samples were then collected from each participant and the sample carefully transported in a viral transport medium (VTM) kept inside an ice pack and taken to the central research laboratory of the College of Medicine, University of Lagos for analysis by polymerase chain reaction (PCR) using consensus primers targeted against the high risk HPV viruses. The samples were screened in pools of five initially for HPV DNA and individual samples from the pool that flagged positive were screened for the high-risk HPV types 16, 18, 31, 33 and 35. QiagenR deoxyribonucleic acid extraction kits was used for the extraction and purification of the viral DNA while amplification of the extracted DNA was carried out using multiplex polymerase chain reaction with the use of specific HPV primers for the high-risk types. Detection of amplified viral DNA was done by agarose gel electrophoresis. All quantitative data were entered in computer and analyzed using SPSS version 20.0 statistical package for windows manufactured by IBM Corp, Armonk, NY, United States. Descriptive statistics were then computed for all relevant data. Bivariate analysis to determine the association between presence of hrHPV infection and the various risk factors was done and all significance were reported at P < 0.05. Ethical approval for the study (HREC Number: ADM/DCST/HREC/2193) was obtained from the hospital's Health Research and Ethics Committee prior to the commencement of the study and the ethical principles according to the Helsinki declaration were considered during the course of the research. All participants read and signed an informed consent form prior to enrolment in the study; the investigators ensured strict confidentiality of all participants' information; The HPV samples were collected and sent for analysis at no cost to the participants and efforts were made to minimise discomfort to the participants during the sample collection and; all participants were given equal attention and optimal care throughout the study and they stand to benefit from the policy that may eventually emanate from the findings of this study.

## Results

**Figure 1:**
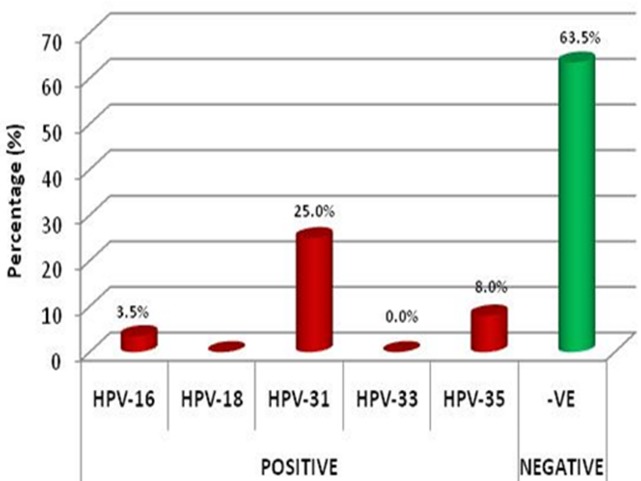
Distribution of tested samples by the predominant high-risk HPV types found

**Figure 2:**
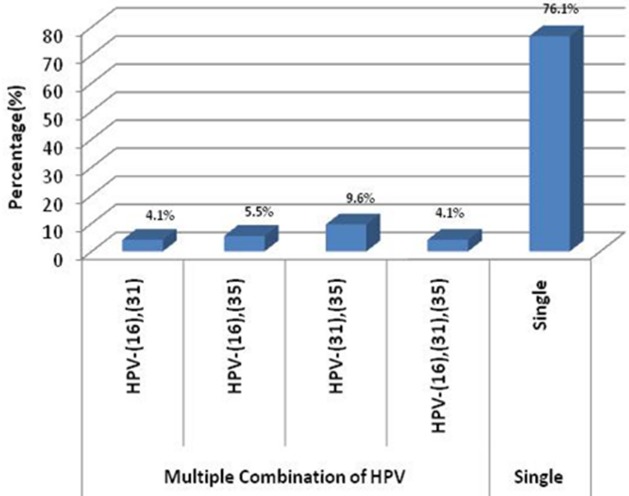
Combination of HPV genotypes in tested samples

A total of 200 women participated in the study with the age range of 20 to 63 years and mean age of 36.1 ± 7.4 years. As shown in Figure 1, 73 (36.5%) of the analyzed samples, were positive for the hrHPV genotypes tested. The commonest HPV type in the positively tested samples was genotype 31 (25.0%) while none (0.0%) of the tested samples was positive for HPV genotypes 18 and 33. In Figure 2, the largest proportion (76.1%) of the tested samples were positive for only a single hrHPV type while the remaining samples have multiple combinations of HPV types with genotypes 31/35 being the commonest (9.6%). The highest proportion of participants with genital hrHPV positivity was in the 30-39 year age group (46.6%). None of the women in the 60-69 years age group had genital HPV. However, there was no statistically significant relationship between the participants' age and hrHPV positivity (P = 0.057) (Table 1). Majority of the women in the study had at least primary level of education (99.0%) but there was no detectable association between the level of education of the recruited women and genital hrHPV positivity (P = 0.852). As shown in Table 2, there was no statistically significant association between the women's parity and genital hrHPV infection (P = 0.664). The largest proportion of the women who had hrHPV in this study had their first child between the age 25 and 29 years (39.7%). There was no detectable genital hrHPV infection in women who had their first child after the age of 34 years. The study, however, found no association between hrHPV infection and age at first delivery (P = 0.705). In Table 3, the study revealed statistically significant associations between genital hrHPV infection and age at coitarche (P = 0.032) and number of lifetime sexual partners (P = 0.001). The study, however, found no association between genital hrHPV positivity and previous oral contraceptive pills usage (P = 0.795); previous Sexually Transmitted Infection (STI) treatment (P = 0.955) or HIV seropositivity (P = 0.063) in the study participants. None of the women with genital hrHPV infection gave a history of having or having had partner(s) who currently has or had died of penile cancer and none of them admitted to current or past history of use of any tobacco product.

**Table 1:** Age and educational status of participants (N = 200)

Characteristics	High-Risk HPV Status		COR (95%CI)	P-value
	Positive	Negative		
	N=73 (%)	N=127 (%)		
**Age group (years)**				
20-29	14 (40.0)	21 (60.0)	1.00(Reference)	0.057
30-39	34 (39.1)	53 (60.9)	0.97 (0.24-3.46)	
40-49	13 (27.7)	34 (72.3)	0.69 (0.11-1.97)	
50-59	11 (42.3)	15 (57.7)	1.06 (0.96-2.01)	
60-69	1 (20.0)	4 (80.0)	0.49 (0.02-6.11)	
Mean age ± SD	36.7 ± 3.9	35.8 ± 1.7		
**Educational status**				
Uneducated	1 (50.0)	1 (50.0)	1.00 (Reference)	0.852
Primary	5 (25.0)	15 (75.0)	0.47 (0.11-5.22)	
Secondary	14 (28.6)	35 (71.4)	0.57 (0.07-3.75)	
Tertiary	40 (39.6)	61 (60.4)	0.79 (0.21-7.24)	
Postgraduate	13 (46.4)	15 (53.6)	0.93 (0.44-7.81)	

*CI=Confidence interval; COR=Crude Odd ratio

**Table 2:** Distribution of participants based on reproductive history (N=200)

Reproductive History	High-Risk HPV Status		COR (95%CI)	P-value
	Positive	Negative		
	N=73 (%)	N=127 (%)		
**Parity**				
0	23 (33.8)	45 (66.2)	1.00 (Reference)	0.664
1-2	31 (36.0)	55 (64.0)	1.07 (1.07-3.88)	
3-4	13 (41.9)	18 (58.1)	1.24 (0.05-6.23)	
>5	6 (40.0)	9 (60.0)	1.18 (0.99-4.16)	
**Age at first delivery (in years)**				
15-19	1 (12.5)	7 (87.5)	1.00 (Reference)	0.705
20-24	19 (38.0)	31 (62.0)	3.04 (2.22-11.65)	
25-29	29 (40.8)	43 (59.2)	3.26 (1.09-8.54)	
30-34	21 (38.9)	33 (61.1)	3.11 (3.07-9.98)	
35-39	2 (15.4)	11 (84.6)	1.23 (0.11-5.03)	
≥40	1 (33.3)	2 (66.7)	2.66 (2.97-8.30)	
Mean age ± SD	27.4 ± 3.3	29.8 ± 5.1		

*CI=Confidence interval; COR=Crude Odd ratio

**Table 3:** Distribution of participants based on sexual history (N=200)

Sexual History	High-Risk HPV Status		COR (95%CI)	P-value
	Positive	Negative		
	N=73 (%)	N=127 (%)		
**Age at coitarche (in years)**				
<25	58 (46.2)	59 (53.8)	1.00 (Reference)	0.032^#^
≥25	15 (18.1)	68 (71.9)	0.39 (0.11-7.76)	
Mean age ± SD	20.1 ± 9.4	24.8 ± 0.3		
**Number of lifetime sexual partners**				
1	13 (21.3)	48 (78.7)	1.00 (Reference)	0.001^#^
2-3	26 (27.9)	67 (72.1)	1.31 (1.10-3.42)	
4-5	11 (32.4)	23 (67.6)	1.52 (0.81-4.44)	
6-7	2 (33.3)	4 (66.7)	1.59 (1.34-4.96)	
>7	1 (50.0)	1 (50.0)	2.34 (2.06-8.57)	
**Use of oral contraceptive pills**				
Yes	21 (37.5)	35 (62.5)	1.00 (Reference)	0.795
No	52 (36.1)	92 (63.9)	0.94 (0.03-5.11	
**Previous treatment of STI**				
Yes	23 (40.4)	34 (59.6)	1.00 (Reference)	0.955
No	50 (34.9)	93 (65.1)	0.86 (0.15-6.01)	
**HIV status**				
Positive	9 (33.3)	18 (66.7)	1.00 (Reference)	0.063
Negative	64 (37.0)	109 (63.0)	1.12 (0.66-2.29)	

*CI=Confidence interval; COR=Crude Odd ratio; STI-sexually transmitted infection

# statistically significant P-value

## Discussion

This study has provided important information about the various risk factors associated with genital hrHPV infection. The reported prevalence of genital hrHPV infection in our study was 36.5%. This is only slightly higher than the prevalence of 30.4% reported by Adegbesan-Omilabu et al in the same city of Lagos [19] but much higher than the 19.7% prevalence found in a study done in Ibadan [6]. This is however much lower than the worldwide prevalence of 10.4% [20] and the rate obtained from other studies done in Asia [21, 22] and North-America [23]. These variations in the prevalent rates may be attributed to the exhaustive nature of the HPV detection strategy used in the various studies especially those carried out in Asia and America and the number of hrHPV types that were tested [19]. The proportion of participants with multiple (two or more) combinations of the hrHPV genotypes detected in the study (23.9%) is higher compared to the multiple infection rate of 8.8% reported Ibadan [24]. The proportional distribution of genital hrHPV genotypes reported in this study was similar to that of a study carried out in Irun (Ondo state), South-West Nigeria in a study involving 13 hrHPV types [25] but it was quite different from the findings of a similar study in Lagos where only a single hrHPV type (type 16) was detected in all the test positive samples [19]. We assumed that the exclusion of women with any form of cervical lesion from this study may explain the lower prevalence of the commonest cervical cancer related-hrHPV genotypes (16 and 18) [16] seen as opposed to genotypes 31 and 35 that we found more predominantly. This finding may be important in the long run on the future designs and implementation of prophylactic HPV vaccines in sub-Saharan Africa.

The finding of the highest proportion of hrHPV infection among the 30 to 39 years age group (46.6%) further corroborated a similar finding in Lagos [19] and Ibadan [24] where older females were found more likely to be infected with hrHPV. Part of the reasons that was proposed for this finding was that a fraction of the spouses or partners of these women may continue to have multiple sexual contacts and thereby re-infecting themselves and these women in the process [24]. However, another study carried Conakry, Guinea showed an increased prevalence of HPV among women who are less than 25 years of age [26]. This study also revealed lack of significant association between the educational level of study participants and genital hrHPV infection. This corroborated the finding in Lagos by Nweke et al [27] and may thus suggest that acquisition of HPV infection which is often thought to be related to sexual lifestyle may not necessarily be influenced by education as tendency to engage in unprotected sexual activities cut across all categories of respondents irrespective of educational status. Our study, however, failed to demonstrate any relationship between genital hrHPV infection and parity, age at first delivery, previous treatment for STI and HIV seropositivity. This finding is mostly at variant with previous studies from within and outside the African continent [12, 15, 19, 21, 23, 27, 28] that showed that parity, use of oral combined oral contraceptive pills; HIV seropositivity and even smoking are all directly or indirectly related to increased sexual activities which may be related to genital hrHPV acquisition. We, however, demonstrated a positive association between hrHPV positivity among the participants and earlier age of sexual debut and increasing number of lifetime sexual partners. The limitation to this study was that it was totally hospital-based and the findings may not representative of the general population. A wider sub-types of hrHPV could have also been studied but for the huge cost implication.

## Conclusion

The prevalence of hrHPV was high in Lagos with majority of test positive samples having only a single HPV genotype. We demonstrated early age of sexual debut and increasing number of lifetime sexual partners as the most important factors associated with genital hrHPV infection. This can, therefore, suggest a need for routine and early screening of these at-risk sexually active women in Nigeria, as well as to further emphasise the importance of sex education for the girl child in school and increase parental awareness towards the HPV vaccination. In view of the diversity of HPV genotypes in our environment, we should also advocate for policy that will ensure the nationwide availability and affordability of the newly designed Nanovalent HPV vaccines through donor assistance programmes and linkage of such programmes with the cervical cancer screening strategies.

### What is known about this topic

- Human papillomavirus (HPV) is now known to be the main factor in the aetiology of cervical cancer with about 100% of cases being associated with previous oncogenic or high-risk human papillomavirus (hrHPV) infection;
- Therefore, since HPV infection is a sexually transmitted infection, cervical cancer is now identified as a sexually transmitted cancer by origin;
- The prevalence of genital human papillomavirus infection in sub-Saharan Africa is considered to be among the highest in the world just like cervical cancer and Nigeria is believed to account for the major proportion of women with this infection.

### What this study adds

- This study determined the current prevalence and distribution of the common genital high risk-HPV infection among Nigerian women attending the out-patient clinics of the Lagos University Teaching Hospital (LUTH);
- The study also provided important information about the various risk factors associated with genital hrHPV infection;
- It thus established a framework to support the activities being implemented currently by planners and decision makers to decrease the burden of cervical cancer in Nigeria and other parts of sub-Saharan Africa through HPV testing and HPV vaccinations.

## Competing interests

The authors declared no competing interests.
